# Entinostat Improves Motor Function and Neuronal Damage *Via* Downregulating NLRP3 Inflammasome Activation After Spinal Cord Injury

**DOI:** 10.3389/fphar.2021.774539

**Published:** 2021-11-26

**Authors:** Chen Dai, Bin Liu, Bibo Peng, Bo Qu, Jiezhi Lin, Baogan Peng, Duan-Ming Li

**Affiliations:** ^1^ Orthopedics and Trauma Department, The 963rd (224th) Hospital of People’s Liberation Army, 963rd Hospital of Joint Logistics Support Force of PLA, Jiamusi, China; ^2^ Department of Orthopedics, The Third Medical Center, General Hospital of the Chinese People’s Liberation Army, Beijing, China; ^3^ Department of Orthopaedics, General Hospital of Northern Theater Command, Shenyang, China; ^4^ Outpatient Department, The Third Medical Center of Chinese People’s Liberation Army General Hospital, Beijing, China; ^5^ Tianjin University, Tianjin Key Laboratory for Disaster and Emergency Medicine Technology, Tianjin, China; ^6^ Military Burn Center, The 963rd (224th) Hospital of People’s Liberation Army, 963rd Hospital of Joint Logistics Support Force of PLA, Jiamusi, China

**Keywords:** SCI, entinostat, HDAC, NLRP3 inflammasome, neuronal damage

## Abstract

**Background:** Spinal cord injury (SCI), a major public health problem, has no effective treatment. A large number of studies have confirmed that histone deacetylases (HDACs) are involved in the physiologic processes that occur following SCI. We tried to uncover the potential neuroprotective role of entinostat (a class I HDAC inhibitor) in SCI.

**Methods:** We conducted a study on a preclinical mouse model of SCI and OGD-induced neuronal damage to present the role of entinostat by the analysis of motor function, histopathologic damage, local NLRP3 inflammasome activation, and neuronal damage.

**Results:** The results showed that entinostat suppressed HDAC activation (including HDAC1 and HDAC3 expression), improved the grip strength and BMS score, spinal edema, cell death, and local NLRP3 inflammasome activation in the spinal cord following SCI. Furthermore, entinostat significantly increased OGD-inhibited neuronal activity and decreased PI-positive cells, HDAC activation, caspase-1 activation, IL-1β and IL-18 levels, and NLRP3 expression.

**Conclusion:** In summary, we first documented that entinostat improved the motor function, histopathologic damage, and local inflammatory response and NLRP3 inflammasome activation in the spinal cord following SCI and also presented the neuroprotective role of OGD-induced neuronal damage *via* the NLRP3 inflammasome. Thus, our study has the potential to reveal the interaction between the HDAC and NLRP3 inflammasome in the pathologic process as well as SCI and further promote the clinical indications of HDACi entinostat and clinical treatment for the inflammatory response after SCI.

## Background

With a high global incidence rate (10.4–83 per million people per year), spinal cord injury (SCI) results in severe long-term disability as it deprives many patients of motor function (Karsy and Hawryluk, 2019), induced by motor vehicle accidents, violence, falls, and recreational activities. In the United States, the incidence of traumatic SCI is 183, 000–230, 000 and is more common in males, with an average age of 31.7 years (McDonald and Sadowsky, 2002), and the average lifetime cost of treating an individual with traumatic SCI is between $1.1 m and $4.7 million per person that places an enormous financial burden on society and families (McDonald and Sadowsky, 2002; Karsy and Hawryluk, 2019). Although a large number of preclinical studies have been conducted to better understand the pathophysiology of acute SCI and to develop targeted therapies to improve SCI outcomes, there is still little clinical practice (Karsy and Hawryluk, 2019). Current clinical management of SCI includes aggressive intensive care measures, early surgical decompression, and stabilization followed by elevating blood pressure to decrease secondary injury (Fehlings et al., 2017; Shank et al., 2019).

Previous research studies have confirmed that damaged cells, axons, and blood vessels release toxic chemicals that attack intact neighboring cells after SCI, and the secondary events include excitotoxicity and inflammation (McDonald and Sadowsky, 2002; Fouad et al., 2021). In animal models of SCI and humans, inflammation is a result of the loss of control of sympathetic tone transmitted to the organs that directs metabolism or mediates immune responses (Fouad et al., 2021). Days or weeks after the initial trauma, a wave of cell death induced by secondary events might sweep through neurons and glial cells affecting the outcomes of the trauma site (McDonald and Sadowsky, 2002). The downstream consequences of inflammation could serve as modifiers of SCI functional outcomes in experimental or clinical indices of the severity of injury (Bloom et al., 2020; Kwiecien et al., 2020; Zrzavy et al., 2021). Several inflammatory treatment strategies have been documented to be effective in preclinical studies of SCI (Jorge et al., 2019; Kong et al., 2021). IL-10 significantly suppresses inflammatory cytokines, alters macrophage phenotype, preserves more axons within the rubrospinal and reticulospinal tracts through the injury site, and aids in functional recovery after SCI (Hellenbrand et al., 2019). Chen et al. have demonstrated that AZD8797, an inhibitor of CX3CR1, effectively blocks overwhelming inflammation, apoptosis, and necrosis after SCI and facilitates early recovery of locomotive function (Chen et al., 2020).

Histone deacetylases (HDACs) are epigenetic regulators that regulate histone modifications, which are also posttranscriptional modifiers that regulate protein acetylation in several pathophysiologic states (Kiany et al., 2020). HDAC inhibitors (HDACi) indicate an ability for targeting two main classes of HDAC enzymes, namely, class I HDAC: 1, 2, 3, and 8 and class II HDAC: 4, 5, 6, 7, 9, and 10 which are utilized in treating diseases ranging from neurodegenerative conditions to inflammatory diseases (Cantley and Haynes, 2013). Multiple HDACi, including trichostatin A (TSA), phenylbutyrate, vorinostat, givinostat, and valproic acid (VPA), have documented anti-inflammatory effects both *in vitro* and *in vivo* (Cantley and Haynes, 2013; Mohammadi et al., 2018). Acetylated histone proteins exert their neuroprotective effects by reducing inflammation and inhibiting neuronal death (Chen et al., 2018), which improve the neurologic functions in many neurologic diseases, including cerebral ischemia (Suda et al., 2015), traumatic brain injury (Sada et al., 2020), and SCI (Su et al., 2014; Chen et al., 2018). VPA inhibits the HDAC3 level and activity and increases STAT1 as well as NF-κB p65 acetylation, which attenuates the microglia-mediated central inflammatory response after SCI (Chen et al., 2018). Zhang et al. verified that the class I HDAC inhibitor CI-994 decreases neutrophil accumulation, inflammatory cytokine expressions, and neuronal loss as early as 3 days following SCI (Zhang H. et al., 2018). Tubastatin A promotes acetylation and stabilization of microtubules and thus restores transport function in nocodazole-treated cells and SCI mice by inhibiting HDAC 6, which may contribute to restored autophagic flux and increased axonal length (Zheng et al., 2020). Hendrix et al. have found specific HDAC 8 inhibition with PCI-34051 reduces the number of perilesional macrophages as measured by histologic analyses but does not improve functional recovery after SCI (Hendrix et al., 2020). Entinostat, a member of the benzamide group, is a narrow-spectrum HDAC inhibitor and affects class I HDAC with limited effect on HDAC 8, which is already used for conducting several phase I/II clinical trials on cancers (Kiany et al., 2020). Furthermore, entinostat has a potential protective effect in ischemic injury, including LPS-induced acute kidney injury by inhibiting ROS oxidative and endoplasmic reticulum stress (Zhang S. et al., 2018) and brain ischemia–induced neuronal death by mitochondrial preservation (Murphy et al., 2014) and NF-κB/RelA-mediated Bcl-xL and Bim levels (Lanzillotta et al., 2013). However, the powerful neuroprotective activity of entinostat in SCI has not been fully understood. Here, we investigated the potential neuroprotective effects of entinostat (15 mg/kg and 45 mg/kg) on improving cognitive impairment and reducing acute spinal damage in an SCI mice model and revealed its potential mechanism in an *in vitro* oxygen-glucose deprivation (OGD)–induced cell model, which may provide a preclinical experimental basis for further elaborating its potential role in the body and SCI clinical therapy.

## Materials and Methods

### The Spinal Cord Injury Model and Entinostat Treatment *in vivo*

Male C57BL/6 mice (25 ± 2 g, 8 weeks) were subjected to SCI. All animal studies were conducted according to the Animal Welfare Guidelines of the United States National Institutes of Health with supervision and ratification from the Animal Care and Use Committee of the General Hospital of the Chinese People’s Liberation Army under standard conditions, including adequate temperature and humidity (60%) and a 12 h light/12 h dark cycle. The mice had free access to water and food.

The SCI model was established as previously described (Jiao et al., 2020). The mice were anesthetized using 4% isoflurane with a rate of 1 L/min, exposed to the T6–T7 spinous process, and underwent laminectomy. Then, epidural compression of the spinal cord with a 24-g closure force was applied for 1 min at the T6–T7 level, resulting in SCI, while the mice that underwent laminectomy were regarded as the sham group. We assessed SCI levels at baseline and postoperative days 1, 2, 3, 7, 14, 21, and 28.

As previously reported (Bombardo et al., 2018; Mota et al., 2020), we dissolved entinostat (Selleck, Shanghai, China) in 0.1 M phosphate-buffered saline (PBS, as the vehicle) (pH 7.4), and it was administered to the animals (1, 5, or 10 mg/kg, gavage) at 4 h after SCI and again 24 and 48 h later. The sham group was treated with equal PBS volumes.

### The Forelimb Grip Strength Detection

The forelimb grip strength was assessed by using a Chatillon digital force meter (LTCM-100, Wintop Co., Shanghai, China). The mice tails were lifted and placed near the bars of a grip-measuring machine so they could grasp the bar. The mice were then gently pulled back horizontally away from the crossbar with a device that measured the grip strength in both front paws. While evaluating the grip of the other front paw, one front paw was taped. If the mouse cannot catch the crossbar, a score of 0 was obtained.

### Basso Mouse Scale

The BMS assessed the hind limb movements during locomotion in an open field, with a 0-to 9-point scale (a high score means better hind limb function). This measurement was conducted independently by two blinded experimenters over 5 min at pre-SCI conditions and 1, 2, 3, 7, 14, 21, and 28 days after SCI (*n* = 8 mice/group).

### Histological Evaluation

The tissues were immersed in 4% paraformaldehyde, dehydrated, and embedded in paraffin. The tissue sections were stained with hematoxylin and eosin (H&E). Microscopic observation of the histologic slides was performed using a light microscope.

### Terminal Deoxynucleotidyl Transferase dUTP Nick-End Labeling Staining

The tissues were obtained, fixed with 4% paraformaldehyde solution, dehydrated, and embedded in paraffin. 4-µm paraffin coronal sections (having proximity to and located in the injury site) were stained by using a DeadEnd™ Fluorometric terminal deoxynucleotidyl transferase–mediated dUTP nick-end labeling (TUNEL) System (Solarbio, Beijing, China, and then incubated with secondary antibodies. The cell nuclei were stained with DAPI (Solarbio). Images were obtained under the fluorescence microscope (Olympus, Osaka, Japan).

### Immunofluorescence Assays

The samples were obtained and fixed with 4% paraformaldehyde, blocked with 5% BSA, and then incubated with primary antibodies, namely, NLRP3 (Thermo Fisher Scientific, Waltham, MA, United States; 1:100) and caspase-1 (Proteintech, 1:100) and then incubated with secondary antibodies. The cell nuclei were stained with 4′,6-diamidino-2-phenylindole (DAPI, Sigma-Aldrich), 6 random high-power fields (200 ×) were chosen, and images were obtained using a fluorescence microscope (Leica, Oskar-Barnack, Germany).

### Primary Neuron Culture and Injury Model *in vitro*

Spinal neurons were obtained from E16 mouse embryos with a modified protocol from Jiao et al. (2020). Briefly, the neonatal pups of C57BL/6 mice were anesthetized by isoflurane, decapitated, and vertebral columns were removed. The spinal cord was isolated and cut into 1-mm^3^ pieces after gently peeling off the meninges. The tissue pieces were digested by 0.25% trypsin for 10 min at 37°C, and then the cells were resuspended in a serum-free medium, including Neurobasal, B27, and penicillin/streptomycin (50 U/mL). 2.5 × 10^5^ cells were seeded in a 24-well plate at 37°C with 5% CO_2_, and supplemented with 10 μM AraC (Sigma-Aldrich, St Louis, MO, United States) after 48 h. On the next day, the medium was replaced with Neurobasal containing supplements, excluding AraC, and changed every three days. The cells were cultured for 10 days and then OGD treatment was performed on them. Briefly, the spinal cord neurons were incubated in DMEM and cultured in an incubator containing 95% N_2_/5% CO_2_ for 4 h. Then, the DMEM was replaced with a standard neuronal culture medium for 24 h. The cells cultured in a standard neuronal culture medium in the presence of ambient 5% CO_2_ served as the control. After OGD treatment, the neurons were either immediately treated with entinostat or not. The vehicle group received an equal volume of PBS.

### Cell Viability Detection

Cell damage was measured using the CellTiter 96^®^ Aqueous One Solution Cell Proliferation Assay (Promega, Madison, WI, United States). The cells were plated in 96-well plates in triplicate at approximately 3 × 10^4^ cells per well and cultured in the growth medium. After treatment, MTS was added to the culture medium and incubated for 2 h at 37°C in 95% humidified air and 5% CO_2_. The absorbance was measured at 490 nm using a microplate reader (Thermo Fisher Scientific).

### Propidium Iodide Staining

After being fixed in 4% paraformaldehyde for 5 min, the treated cells were washed thrice with PBS and then stained with 500 nM of propidium iodide (PI; Sigma-Aldrich) for 5 min. DAPI (Sigma-Aldrich) was used to stain nuclei, and images were obtained with a fluorescence microscope.

### ELISA Analysis

The spinal tissues and the cell culture medium supernatant were collected. A bicinchoninic acid (BCA) assay kit (Invitrogen, Carlsbad, CA, United States) was used to measure the protein concentration. ELISA analysis was performed using the TNF-α, IL-1β, and IL-18 ELISA kit (Beyotime Biotechnology, Shanghai, China) following the manufacturer’s instructions.

### Caspase-1 Activity Detection

Caspase-1 activity was detected using the Caspase-1 Activity Assay Kit (Solarbio). The specific steps were performed according to the manufacturer’s instructions (Solarbio). The protein concentration was ensured from 1 μg/μl to 3 μg/μl. A standard curve was prepared using the pNA standard. Then, the optical density of the specimen was read on a microplate reader (Thermo Fisher Scientific) at 405 nm. The percentage of changes in caspase-1 activity was calculated by the ratio of OD_405_ of the experimental well to that of the normal well.

### Histone Deacetylase Activity Analysis

HDAC enzyme activity was conducted by the EpiQuik HDAC Activity/Inhibition Assay Kit (Colorimetric) (Epigentek, Farmingdale, NY, United States). Briefly, nuclear cell lysates were extracted using the Nuclear Protein Extraction Kit (Solarbio), 50 µg of the lysates was incubated with the HDAC colorimetric substrate, and the color was immediately quantified through an ELISA reaction. HDAC activity was inversely proportional to the amount of the remaining acetylated histone substrates. HDAC activity or inhibition was calculated using the following formula: HDAC activity (OD/min/mg) = ((Sample OD–Blank OD)/ (Protein Amount (50 µg) ×60 min)) × 1,000.

### Western Blot Analysis

Spinal cord tissue and primary neurons were lysed by lysis buffer (Beyotime, Shanghai, China). After protein quantification, electrophoresis (12% SDS-PAGE gels), and Western transfer, the membranes were blocked with 5% BSA and incubated with the appropriate primary antibodies, such as HDAC1 (CST, 1:1,000), HDAC2 (Abcam, 1:800), HDAC3 (Abcam, 1:1,000), NLRP3 (Thermo Fisher Scientific, 1:1,000), caspase-1 (Proteintech, 1:800), and caspase-1 p20 (CST, 1:1,000) at 4°C overnight and then incubated with secondary antibodies (Abgent, 1: 20,000) followed by the hypersensitivity chemiluminescent substrate (Bio-Rad, Hercules, CA, United States), and protein bands were photographed by using a ChemiDoc™ MP imaging system (Bio-Rad).

### QPCR Analysis

Total RNA was lysed by using the TRIzol reagent (Invitrogen) and reversely transcripted to cDNA using a HiFi-MMLV cDNA First-Strand Synthesis Kit (CW-Bio, Beijing, China). QRT-PCR analysis was executed using GoTaq qPCR Master Mix (Tiangen, Beijing, China) by the CFX96TM Real-Time System (Bio-Rad, Hercules, CA, United States). QPCR was performed on samples and standards in triplicate, and fold changes were calculated by applying the relative quantification (2^−ΔΔCt^) method. GAPDH was used as an internal control, and primer sequences are listed as follows.

**Table:** 

HDAC1	Forward	5′-TGA​TGC​TGG​GAG​GAG​GTG-3′
	Reverse	5′-GTT​GGA​AGG​GCT​GAT​GTG-3′
HDAC2	Forward	5′-TGA​CAA​ACC​AGA​ACA​CTC​CAG​AAT​A-3′
	Reverse	5′-GAA​TAG​CTT​GCA​TTT​GAA​CAC​CAG-3′
HDAC3	Forward	5′-AGC​CTT​AAT​GCC​TTC​AAC​GTG​G-3′
	Reverse	5′-TCA​TTG​ACA​TAG​CAG​AAG​CCA​GAG​G-3′
HDAC4	Forward	5′-GCG​AGC​ACA​GAG​GTG​AAG​ATG​AAG-3′
	Reverse	5′-AGA​CGG​GGT​GGT​TGT​AGG​AGG-3′
HDAC6	Forward	5′-TGG​TGT​TAT​GTC​TGT​CAG​GCT​TA-3′
	Reverse	5′-GCA​GTG​TGG​TCT​GGG​ATT​TAG​T-3′
HDAC8	Forward	5′-TGT​GAC​TCC​CTG​GCC​AAG​ATC​CC-3′
	Reverse	5′-TCA​TCG​CCC​TCT​TGG​CTG​ACC​TT-3′
HDAC11	Forward	5′-GTT​TAC​AAC​CGC​CAC​ATC​TAC​CC-3′
	Reverse	5′-TCC​ACC​TTC​TCC​AGA​TAT​TCC​TCA​T-3′
GAPDH	Forward	5′- AAC​TTT​GGC​ATT​GTG​GAA​GG -3′
	Reverse	5′- GGA​TGC​AGG​GAT​GAT​GTT​CT -3′

### Statistical Analysis

Data were expressed as the mean ± SEM. SPSS 22.0 was used for Student’s *t*-test or one-way ANOVA followed by Tukey’s multiple comparisons test (two-way ANOVA analysis for grip strength assessment). *P* < 0.05 was considered statistically significant.

## Results

### Entinostat Improves the Motor Function Following Spinal Cord Injury

**FIGURE 1:**
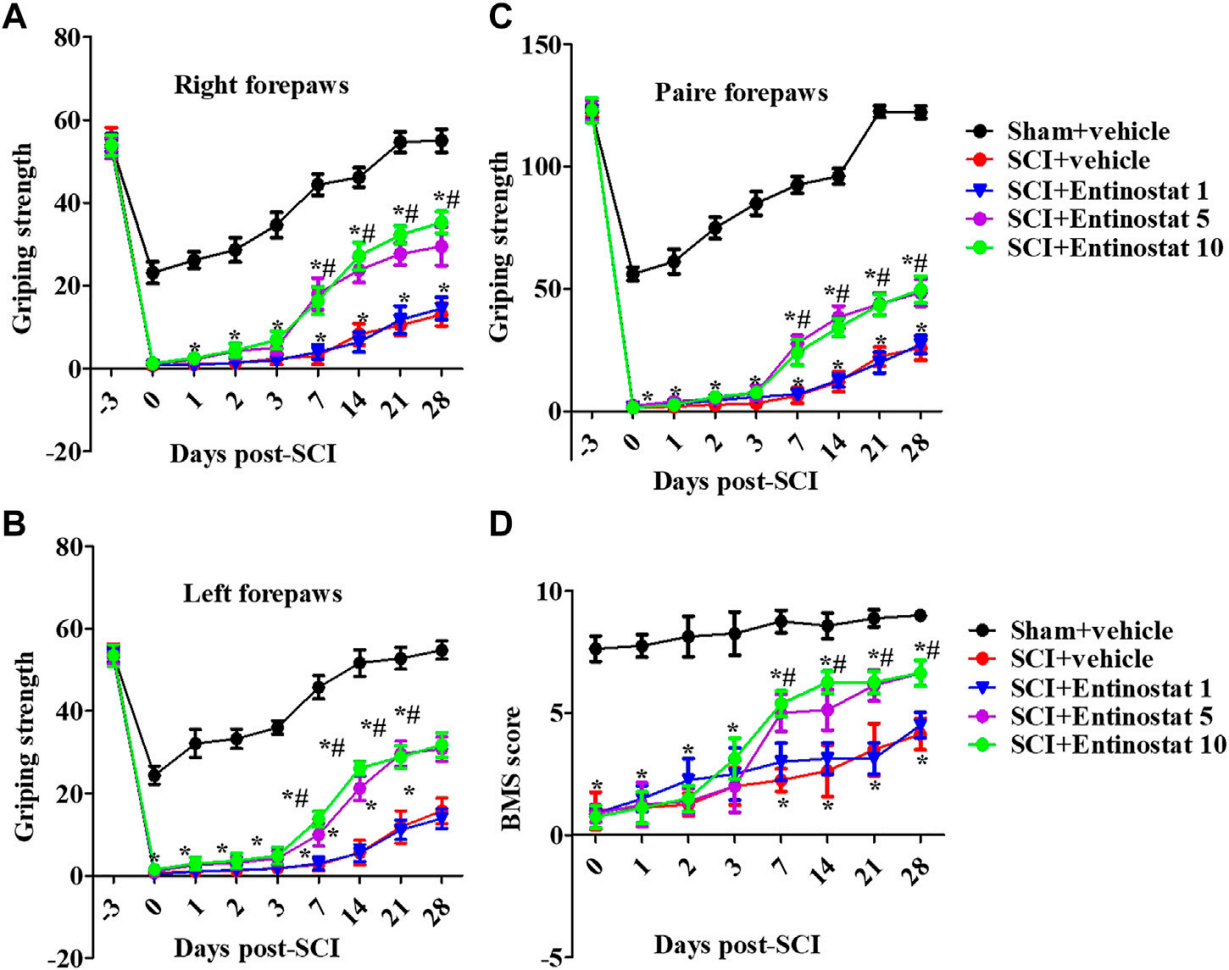
Entinostat improves the motor function following SCI. Behavioral tests of SCI mice with entinostat (1, 5, or 10 mg/kg) or not and the grip strength of the right forepaw **(A)**, left forepaw **(B)**, and paired forepaws **(C)** was measured before the experiment and after surgery. **(D)** Measurement of the BMS score after SCI. Data are expressed as the mean ± SEM, *n* = 8. *p < 0.05, *vs.* the sham + vehicle group; #*p* < 0.05, *vs.* the SCI + vehicle group. BMS: basso mouse scale. SEM: standard error of the mean.

After the onset of SCI within 4 weeks, the motor function of mice was assessed by the forelimb grip strength test and BMS score. Before the experiment, all the mice showed consistent behavioral evaluations. SCI directly impaired motor function with decreased grip strength both of left, right, and paired forepaws (Figures 1A–C), and the grip strength had a slow recovery process during the 1 week following SCI. Entinostat improved the motor function in a concentration-dependent manner, at 7–28 days after SCI (but not during the 7 days following SCI) (Figures 1A–C), where 5 mg/kg and 10 mg/kg of entinostat showed significant protection of motor dysfunction (not the 1 mg/kg group) (*p*<0.05, Figures 1A–C), but there was still a gap compared with the characteristics in the sham group (Figures 1A–C). At 7–28 days after SCI, the BMS score was significantly improved in the entinostat 5 mg/kg and 10 mg/kg groups than the sham group (*p*<0.05, Figure 1D). But, the entinostat 1 mg/kg group at 28 days post-SCI showed a statistically significant BMS score alteration compared with that of the sham group (*p*<0.05, Figure 1D). The data showed that high concentrations of entinostat (5 mg/kg and 10 mg/kg) treatment presented the protection of motor function following SCI.

### Entinostat Attenuates Histopathologic Damage Following Spinal Cord Injury

**FIGURE 2:**
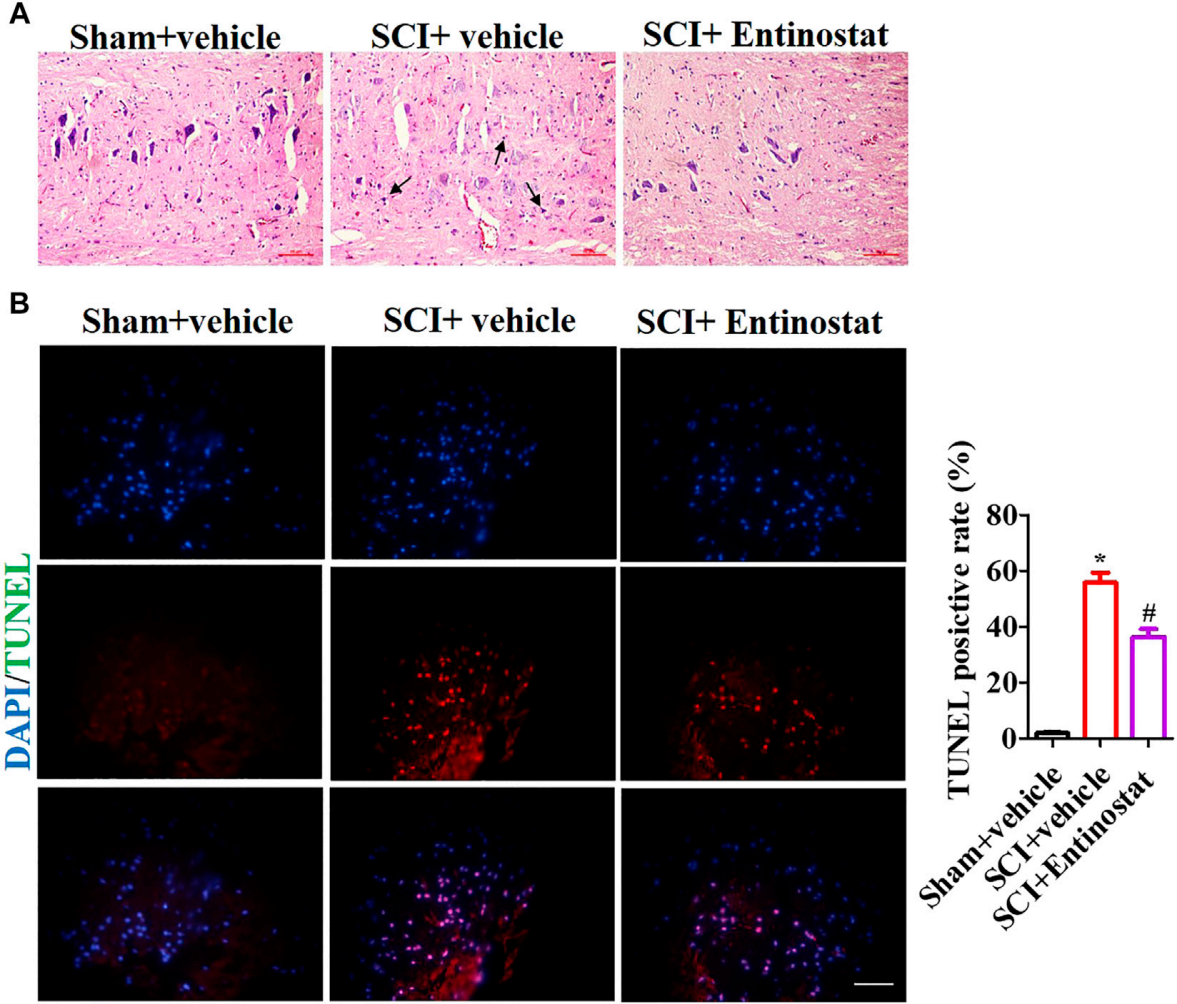
Entinostat attenuates histopathologic damage following SCI. The histopathologic damage was assessed 7 days following SCI by H&E staining. **(A)** Black arrows indicating the neutrophil infiltration and TUNEL staining. **(B)** Nuclei were stained with DAPI. Histogram analysis of the TUNEL-positive cell. Data are expressed as the mean ± SEM, *n* = 6. Scale bar: 25 μm *p < 0.05, *vs.* the sham + vehicle group; #*p* < 0.05, *vs.* the SCI + vehicle group.

To explore the protection of entinostat on histopathologic damage, H&E and TUNEL staining were performed to assess the pathologic alteration after treating with 5 mg/kg entinostat. The H&E staining results showed that the structure of the spinal cord was clear, the nucleus was complete and inerratic, and there was hypochromatic nucleus, neutrophil infiltration (as the black arrow indicating), neuronal disruption, and diffuse hemorrhage (Figure 2A). However, entinostat relieved histopathologic damage after SCI (Figure 2A). By TUNEL staining, the results found more TUNEL-positive cells (about 64.3%) in the SCI group compared with the sham group, and entinostat treatment significantly suppressed cell death (about 27.5% TUNEL positive cell) (Figure 2B). These results indicated that entinostat attenuates histopathologic damage and showed protection post-SCI.

### Entinostat Inhibits NLRP3 Inflammasome Activation After Spinal Cord Injury

**FIGURE 3:**
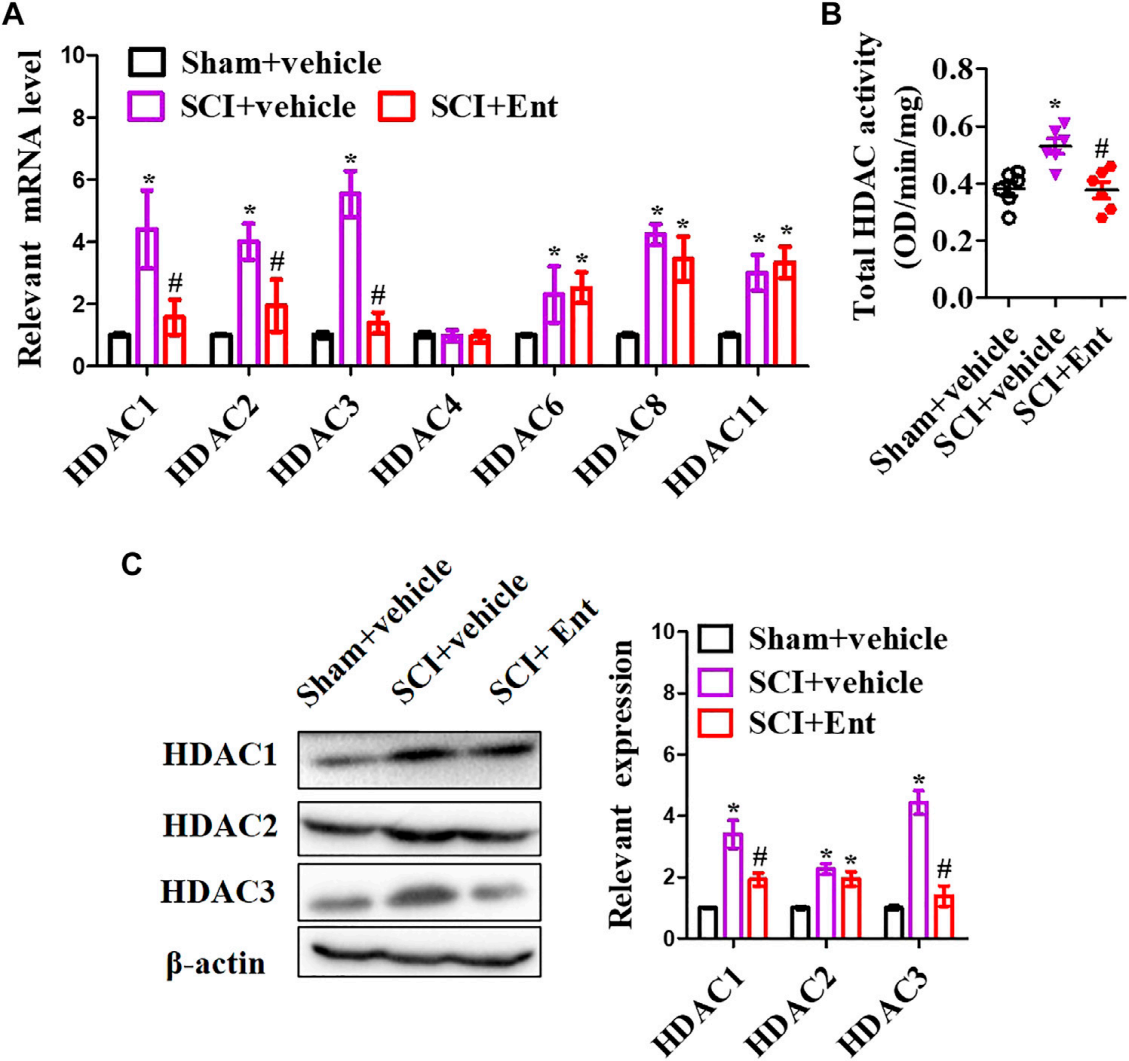
Entinostat suppresses HDAC1 and HDAC3 expressions in the spinal cord after SCI. **(A)** 7 days following SCI, HDAC1, HDAC2, HDAC3, HDAC4, HDAC6, HDAC8, and HDAC11 mRNA concentrations were measured. **(B)** Enzymatic activity of total HDAC was evaluated using the EpiQuik HDAC Activity/Inhibition Assay Kit. **(C)** WB analysis of HDAC1, HDAC2, and HDAC3 expressions in the spinal cord following SCI at 7 days. Data are expressed as the mean ± SEM, *n* = 5. *p < 0.05, *vs.* the sham + vehicle group; #*p* < 0.05, *vs.* the SCI + vehicle group.

**FIGURE 4:**
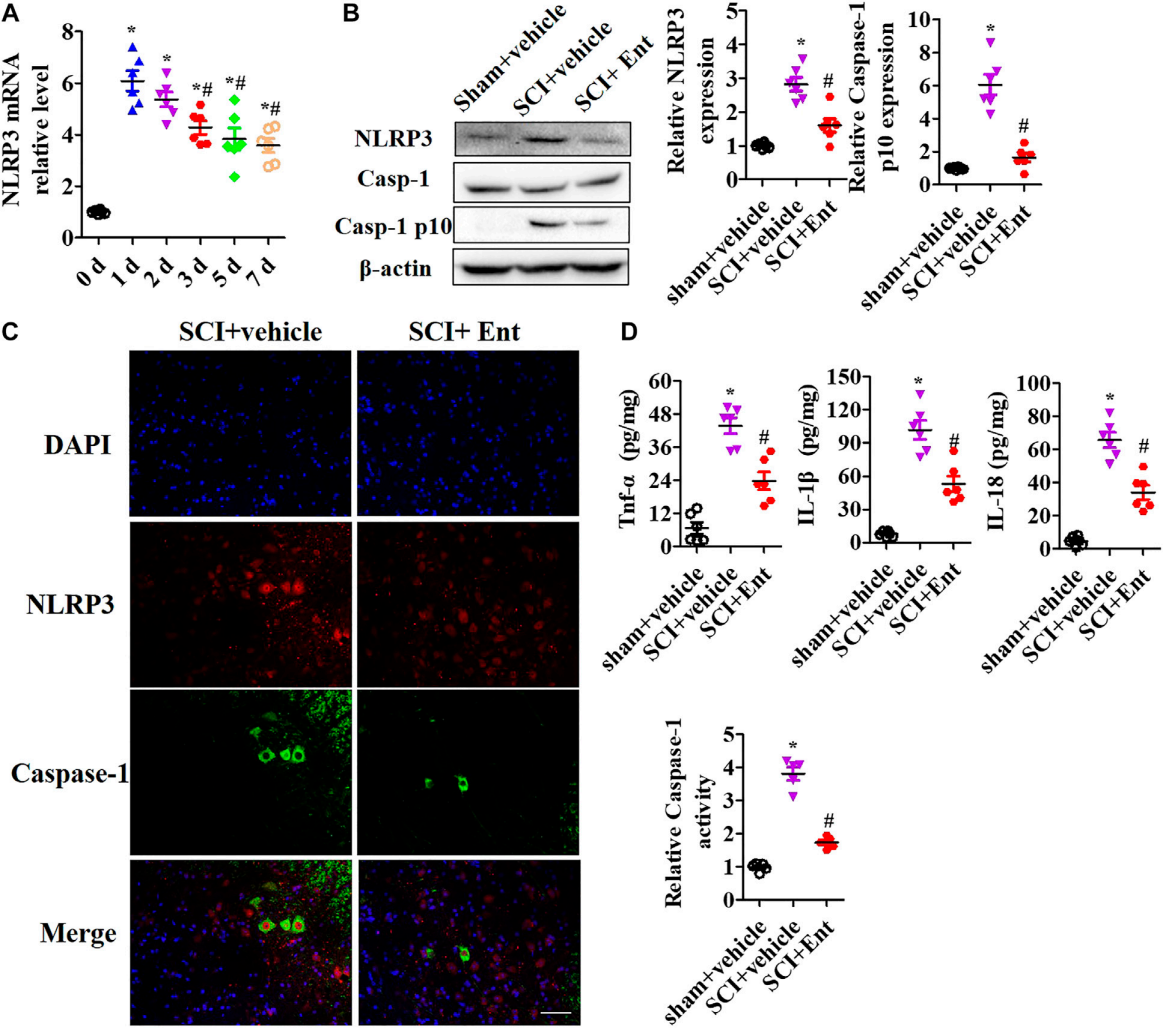
Entinostat inhibits NLRP3 inflammasome activation in the spinal cord after SCI. NLRP3 inflammasome activation was measured in the spinal cord after SCI. **(A)** qPCR analysis of NLRP3 mRNA during 7 days post-SCI. **(B)** WB analysis of NLRP3, Caspase-1 expression in spinal cord after SCI at 7 days. Histogram analysis of the protein relative expression, *vs.* β-actin. **(C)** NLRP3/caspase-1 double staining was performed, and the nuclei were stained with DAPI. Scale bar: 50 μm. **(D)** ELISA analysis of the levels of TNF-α, IL-1β, and IL-18 in the spinal cord. **(E)** Caspase-1 activity was detected using Caspase-1 Activity Assay Kit after SCI. Data are expressed as the mean ± SEM, *n* = 5. *p < 0.05, *vs.* the sham + vehicle group; #*p* < 0.05, *vs.* the SCI + vehicle group.

After SCI at 7 days, HDACs were measured. As shown in Figure 3A, SCI increased class I HDAC levels, including HDAC1, HDAC2, HDAC3, HDAC6, HDAC8, and HDAC11 mRNA, while entinostat significantly suppressed SCI-induced class I HDAC levels (*p* < 0.05, Figure 3A). Total HDAC activation was also upregulated after SCI and decreased after entinostat treatment (*p* < 0.05, Figure 3B). WB results showed that the expressions of HDAC1, HDAC2, and HDAC3 were induced by SCI in the spinal cord and entinostat inhibited the expressions of HDAC1 and HDAC3, but the variation in HDAC2 expression was weak following treatment with entinostat (Figure 3C). The data showed that class I HDAC involved in the pathologic processes of SCI and entinostat suppressed HDAC1 and HDAC3 expression in the spinal cord. Furthermore, we analyzed NLRP3 inflammasome activation after SCI. As shown in Figure 4A, NLRP3 mRNA was increased with time dependence, which peaked at 24 h following SCI, then decreased gradually from 2 to 7 days, but was significantly upregulated at 7 days (*p* < 0.05, Figure 4A). Then, NLRP3 expression was measured at 7 days post-TBI in the spinal cord of entinostat-treated mice. Undoubtedly, SCI not only induced NLRP3 expression and caspase-1 p 10 fragment but also was significantly suppressed by entinostat compared with the SCI group (*p* < 0.05, Figure 4B). IF results demonstrated that entinostat inhibited NLRP3/caspase-1 staining (Figure 4C). Furthermore, NLPR3 inflammasome–mediated inflammation was also measured in the spinal cord. ELISA results showed that SCI increased the level of inflammatory factors, such as TNF-α, IL-1β, and IL-18 at 7 days post-SCI, whereas entinostat significantly decreased the levels of TNF-α (*p*<0.05), IL-1β (*p*<0.01), and IL-18 (*p*<0.01) (Figure 4D). Caspase-1 activity assay also demonstrated that entinostat reversed the increase of caspase-1 activity after SCI (*p*<0.01, Figure 4E). Thus, the results demonstrated that entinostat alleviated NLPR3 inflammasome activation in the spinal cord following SCI.

### Entinostat Ameliorates NLPR3 Inflammasome Activation and Neuronal Damage

**FIGURE 5:**
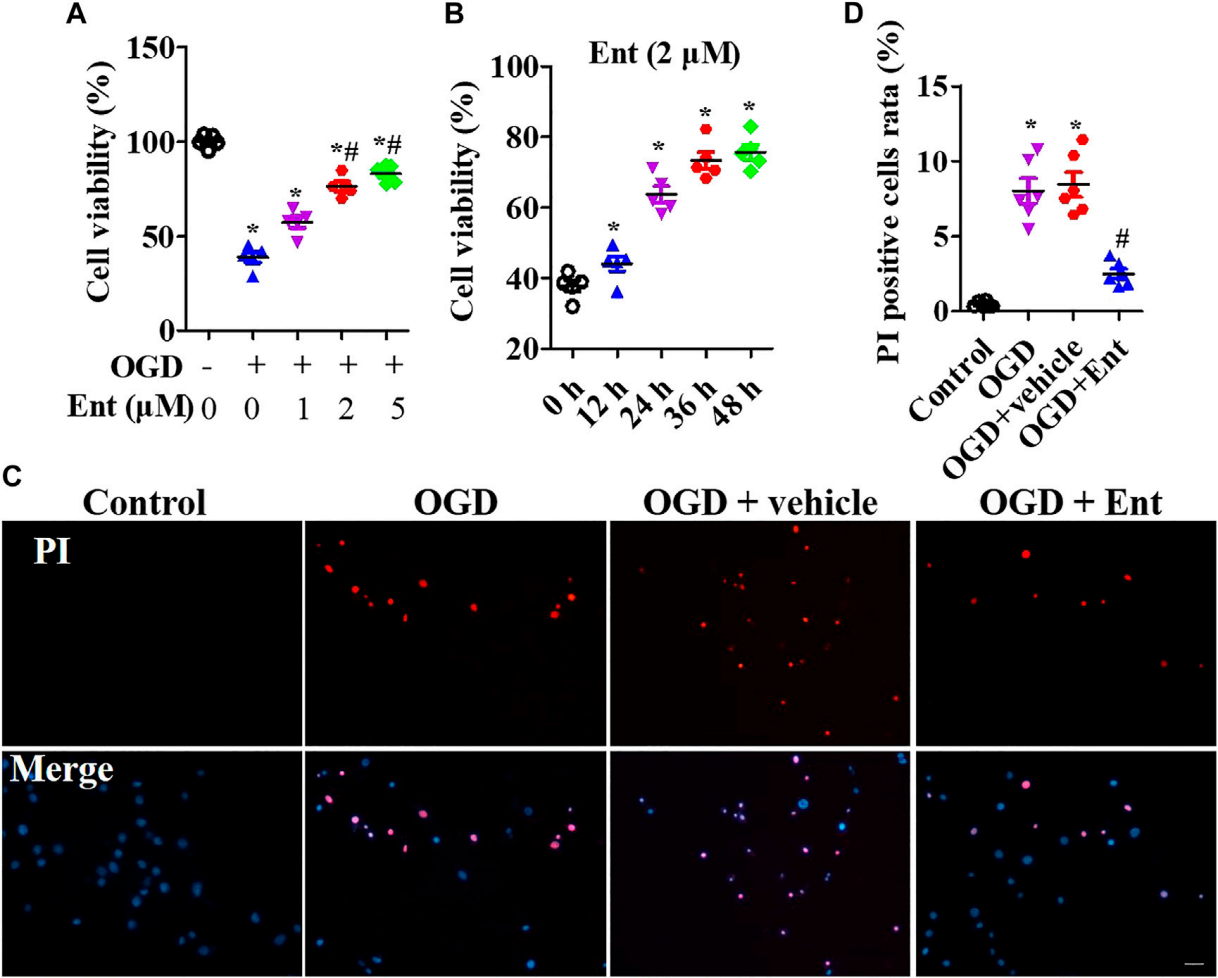
Entinostat suppresses OGD-induced neuronal injury. In the OGD-induced neuronal damage model, cell viability was measured after being treated with 0, 1, 2, or 5 μM entinostat for 24 h **(A)** or treated with 2 μM entinostat for 12, 24, 36, or 48 h **(B)**. **(C)** PI staining of primal neurons at 24 h after being treated with 2 μM entinostat, and DAPI stained all cell nuclei. **(D)** Histogram analysis of PI-positive cells. Scale bars: 50 μm. Data are expressed as the mean ± SEM, *n* = 4. *p < 0.05, *vs.* the control group; #*p* < 0.05, *vs.* the OGD group.

**FIGURE 6:**
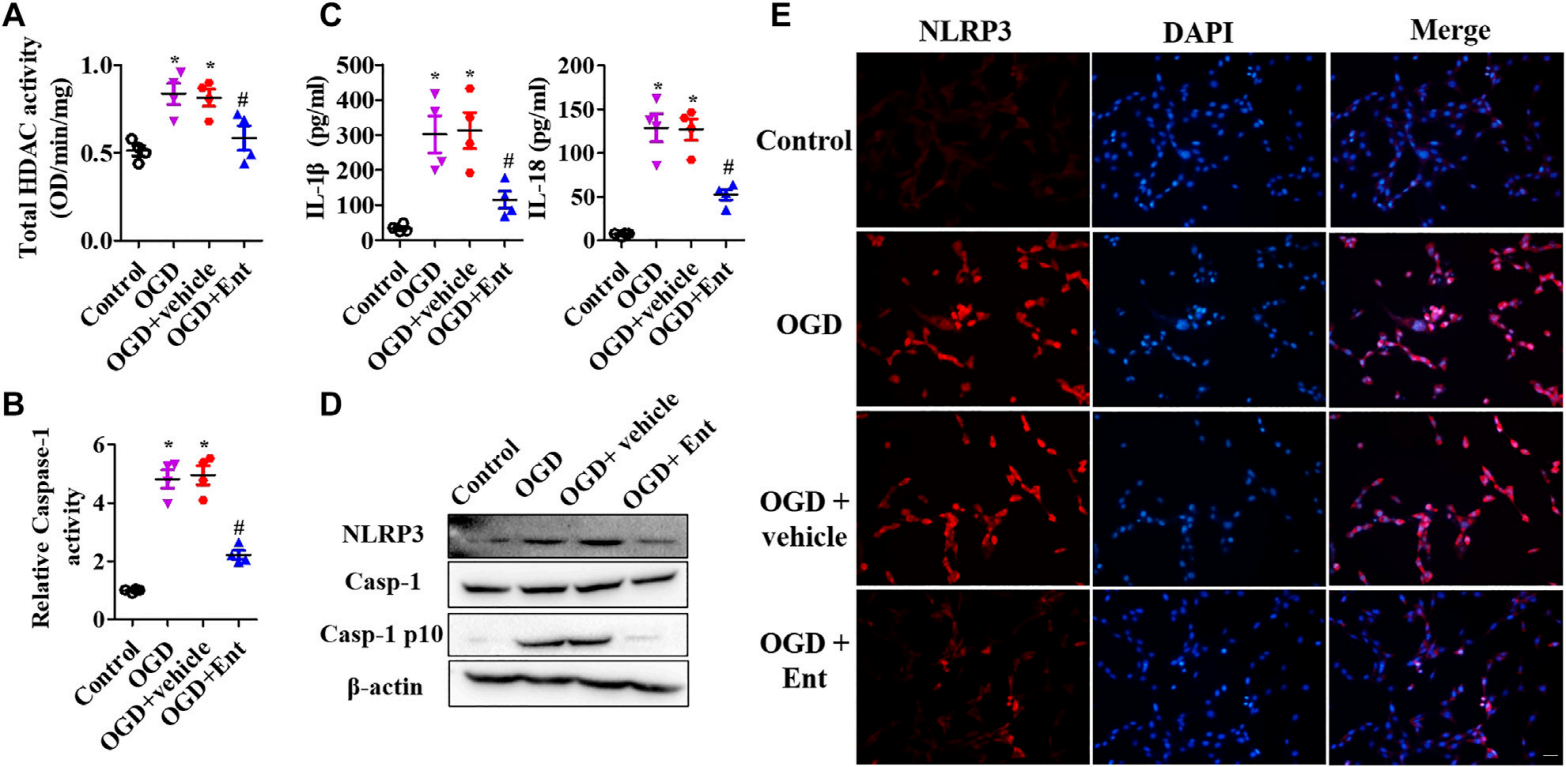
Entinostat ameliorates OGD-induced NLPR3 inflammasome activation. In the OGD-induced neuronal damage model, enzymatic activity of total HDAC was evaluated using the EpiQuik HDAC Activity/Inhibition Assay Kit **(A),** and caspase-1 activity was detected using Caspase-1 Activity Assay Kit **(B)**. **(C)** ELISA analysis of the levels of IL-1β and IL-18 in primal neurons. **(D)** WB analysis of NLRP3, caspase-1, and caspase-1 p 10 levels. **(E)** IF analysis of NLRP3 expression in the OGD model, Scale bar: 50 μm. Data are expressed as the mean ± SEM, *n* = 4. *p < 0.05, *vs.* the control group; #*p* < 0.05, *vs.* the OGD group.

MTS was used to detect the cell viability of primal neurons. At the beginning of OGD, spinal cord neurons were immediately treated with entinostat (1, 2, and 5 μM) for 24 h or not. As shown in Figure 5A, OGD downregulated cell viability, and high concentration of entinostat (2 and 5 μM) could inhibit the decrease of cell activity, but the effect of 1 μM entinostat was weak (Figure 5A). Then, 2 μM entinostat was treated for 12, 24, 36, and 48 h. The longer the treatment time of entinostat, the better the cell activity of neurons (Figure 5B). So, the treated condition of entinostat was selected as 2 μM for 24 h. As shown in Figures 5C,D, PI staining showed that OGD induced the increase of PI-positive cells, and 2 μM entinostat significantly decreased the PI-positive cells. After entinostat treatment in OGD-induced primal neurons, total HDAC activation and caspase-1 activation were analyzed at 24 h. OGD significantly enhanced the total HDAC (*p*<0.05, Figure 6A) and caspase-1 (*p*<0.05, Figure 6B) activation, and entinostat reduced the total HDAC and caspase-1 activation (*p*<0.05, Figures 6A,B). ELISA results showed that OGD increased the level of inflammatory factors, IL-1β and IL-18, whereas entinostat significantly decreased the levels of IL-1β and IL-18 (*p*<0.05, Figure 6C). WB results further found that entinostat significantly decreased HDAC3 expression and NLRP3 inflammasome activation (including NLRP3 expression and caspase-1 p 10 level), related to the OGD group (*p*<0.05, Figure 6D). Furthermore, IF results showed that OGD induced NLRP3 expression and entinostat suppressed NLRP3 expression in the OGD model (Figure 6E). The results demonstrated that entinostat alleviated NLPR3 inflammasome activation and neuronal damage in the OGD model.

## Discussion

Entinostat is a narrow-spectrum HDAC inhibitor of class I HDAC that has been shown to possess neuroprotective functions in brain ischemia and intracerebral hemorrhage (Mota et al., 2020; Bonsack and Sukumari-Ramesh, 2021). However, its role in SCI has not been reported. In this study, we showed that entinostat improved the motor function (including the grip strength and BMS score), histopathologic damage (spinal edema and cell death), and local NLRP3 inflammasome activation in the spinal cord following SCI. Furthermore, entinostat significantly increased OGD-inhibited neuronal activity and decreased PI-positive cell concentration, HDAC activation, caspase-1 activation, IL-1β and IL-18 levels, and NLRP3 expression.

A large number of studies have confirmed that HDACs are involved in the physiologic processes that occur following SCI (Zhang H. et al., 2018; Chen et al., 2018; Hendrix et al., 2020; Zhou et al., 2020). Recent studies have shown that HDACs tend to increase in PBMC nuclear extracts of SCI patients, including increased HDAC3 in PBMCs from SCI patients (Ma et al., 2015). In the animal SCI model, the HDAC expression is not consistent. Chen et al. documented that HDAC 1–3 is upregulated in the lesioned spinal cord of male Wistar rats 7 days after SCI, when compared to the sham group (Chen et al., 2018). The treatment with VPA inhibits HDAC3 protein expression and induced STAT1, as well as NF-κB p65 acetylation following SCI, without affecting the HDAC1 and the HDAC2 protein expressions, which attenuates the microglia-mediated central inflammatory response after SCI (Chen et al., 2018). Zhou et al. also verified that upregulated HDAC3 is presented in the spinal cord tissues of experimental SCI SD rats and HDAC3 knockdown restores the locomotor function via HDAC3/miR-27a/PAK6 axis (Zhou et al., 2020). Interestingly, Zhang et al. have shown that among class I HDACs, HDAC1 and HDAC3 expressions are significantly downregulated in the motor cortex of C57BL/6 J mice but not significantly altered in the lesion site of the spinal cord 14 days after SCI. No expression changes in HDAC1 and HDAC3 were measured in the spinal cord of CI-994-treated mice (Zhang S. et al., 2018). The administration of CI-994 inhibits class I HDAC activities in both the cortex and spinal cord, and CI-994 enhances functional recovery following SCI but does not enhance cervical sprouting of the corticospinal tract following SCI (Zhang H. et al., 2018). Although Zhang et al. showed that the variation trend of HDACs in the spinal cord of the SCI model is inconsistent with previous reports, they also revealed that the inhibition of both HDAC1 and HDAC 3 of CI-994 may exert a neuroprotective effect (Zhang S. et al., 2018). In our study, we found that HDAC1, HDAC2, and HDAC3 expressions were enhanced in the spinal cord after SCI and entinostat decreased HDAC1 and HDAC3 expressions but without affecting the HDAC2 expression. Entinostat, another class I HDAC inhibitor, has potential neuroprotective effects in brain damage (Zhang H. et al., 2018; Hendrix et al., 2020; Bonsack and Sukumari-Ramesh, 2021). Entinostat reduces NF-κB-p65 expression and nuclear accumulation as well as the neuroinflammatory response (TNF-α and IL-1β levels) in the hippocampus, which causes the improvement of postoperative cognitive dysfunction in rats (Wu et al., 2019). Local infusion of entinostat into the medial prefrontal cortex exerts robust antidepressant-like effects in the chronic social defeat stress paradigm in mice (Covington et al., 2015). Ma et al. documented that entinostat treatment contributes to the recovery of actin-based NMDAR synaptic delivery and the rescue of autistic social preference deficits in Shank 3–deficient mice (Ma et al., 2018). Finelli et al. reported that entinostat treatment enhances the expression of genes associated with axon regeneration in sensory neurons and promotes axonal growth after SCI (Finelli et al., 2013). Furthermore, our experiment results first suggested the potential role of entinostat in the spinal cord that entinostat improved histopathologic damage and motor function of male C57BL/6 mice following SCI and showed the neuroprotective role of OGD-induced neuronal damage.

Current investigations have demonstrated that NLRP3 inflammasome–mediated cell death plays a critical role in the pathogenesis of multiple neurologic disorders (Heneka et al., 2018; Voet et al., 2019; Chen et al., 2021), as well as SCI (Jiang et al., 2017; Al Mamun et al., 2020). NLRP3 inhibitors play a neuroprotective role in SCI (Jiao et al., 2020), as well as the NLRP3 inflammasome upstream regulatory molecules, such as ROS, NF-Κb, NEK7, and ASC (Gao et al., 2019; Shiraishi et al., 2020; Ji et al., 2021). In SCI mouse models, NEK7 suppression attenuates local inflammatory response and inhibits NLRP3 inflammasome activation in microglia/macrophages of the injured spinal cord, and targeting the NEK7/NLRP3 signaling shows great promise in the treatment of inflammatory responses after SCI (Ji et al., 2021). In addition, several potential drugs also present a neuroprotective role by regulating NLRP3 activation (Gao et al., 2019; Kong et al., 2021). The administration of echinacoside enhances the BBB scores, decreases neuron loss, and improves tissue architecture via inhibiting the NLRP3 inflammasome signaling pathway after SCI (Gao et al., 2019). β-hydroxybutyrate inhibits an LPS + ATP-induced inflammatory response and NLRP3 levels, and a ketogenic diet attenuates neuroinflammation following SCI, probably by suppressing the NLRP3 inflammasome and shifting the activation state of microglia from M1 to the M2a phenotype (Kong et al., 2021). At the same time, we also demonstrated that entinsotat inhibited the local inflammatory response and NLRP3 inflammasome activation after SCI, which might explain its contribution to the improvement of motor function. Previously, correlations between HDAC and NLRP3 have been rarely reported. Ky-2, a hybrid compound HDACi, downregulates LPS-induced NLRP3, caspase-1 p 20, and IL-1β in THP-1 cells that may regulate M1 macrophage polarization via inhibiting NLRP3 inflammasome activation (Kaneko et al., 2018). In J774A.1 cells, hydroxamic acid derivatives of nigranoic acid and manwuweizic acid moderately enhance HDAC1/2/4/6 inhibition activity, which inhibits IL-1β maturation and caspase-1 cleavage, and Ni et al. suggest that the synthesis of HDACi may block NLRP3 inflammasome activation (Ni et al., 2021).

## Conclusion

In conclusion, we first documented that entinostat improved motor function, histopathologic damage, local inflammatory response, and NLRP3 inflammasome activation in the spinal cord following SCI and also presented the neuroprotection of OGD-induced neuronal damage via the NLRP3 inflammasome. Thus, our study has the potential to reveal the interaction between the HDAC and NLRP3 inflammasome in the pathologic process as well as SCI and further promote the clinical indications of HDACi entinostat and clinical treatment for inflammatory response after SCI.

## Data Availability

The original contributions presented in the study are included in the article/Supplementary Material; further inquiries can be directed to the corresponding authors.
